# Diagnosis of Behavioral Symptoms of Dementia and CNS-Active Drug Use Among Diverse Persons Living With Dementia

**DOI:** 10.1093/geroni/igab046.1055

**Published:** 2021-12-17

**Authors:** Johanna Thunell, Geoffrey Joyce, Dima Qato, Jenny Guadamuz, Julie Zissimopoulos

**Affiliations:** 1 University of Southern California, Los Angeles, California, United States; 2 University of Southern California, University of Southern California, California, United States

## Abstract

Approximately 90% of persons living with dementia (PLWD) experience behavioral and psychological symptoms of dementia (BPSD). Studies demonstrated high use of central nervous system (CNS) active drugs in nursing homes; one recent study documented high use among community-dwelling PLWD. Racial/ethnic disparities in BPSD diagnosis and CNS-active drug use, however, are unknown. We quantified disparities in BPSD diagnoses and CNS-active drug use using 100% Medicare Part A and B claims, 2017-2019, and Part D, 2018-2019. Beneficiaries were ages 65 and older in 2017, community-dwelling, and had a dementia diagnosis (n=801,597). We estimated models of CNS-active drug use to quantify racial/ethnic differences adjusting for confounders. Among PLWD, 66% had a BPSD diagnosis and 65% were taking a CNS-acting drug. Asians/Pacific Islanders were less likely to have a BPSD diagnosis (55%) than other groups, particularly affective diagnoses (40%). Whites were most likely to have any diagnosis (67%). Blacks were most likely to have hyperactivity diagnoses (7%). Antidepressants were most commonly used drug class (44%). Thirteen percent used an antipsychotic. Models adjusted for age, sex, comorbid conditions, dual-eligibility and BPSD diagnoses, showed non-Whites were less likely to use any CNS-active drug than Whites, but Blacks and Hispanics were slightly more likely to use antipsychotics. We found racial/ethnic differences in BPSD diagnoses and CNS-active drug use. Whether these disparities are due to differences in BPSD symptoms, health-care access or care-seeking remains an important question. Further study of disparity in outcomes associated with use will inform risk and benefit of CNS-active drugs use among PLWD.

